# Influence of mobile genetic elements and insertion sequences in long- and short-term adaptive processes of *Acidithiobacillus ferrooxidans* strains

**DOI:** 10.1038/s41598-023-37341-4

**Published:** 2023-07-05

**Authors:** Ana Moya-Beltrán, Martin Gajdosik, Camila Rojas-Villalobos, Simón Beard, Martin Mandl, Danitza Silva-García, D. Barrie Johnson, Pablo Ramirez, Raquel Quatrini, Jiri Kucera

**Affiliations:** 1 Fundación Ciencia & Vida, Avenida Del Valle Norte 725, 8580702 Huechuraba, Santiago Chile; 2 Facultad de Ingeniería, Arquitectura y Diseño, Universidad San Sebastián, Santiago, Chile; 3 Department of Biochemistry, Faculty of Science, Masaryk University, 61137 Brno, Czech Republic; 4 Facultad de Medicina y Ciencia, Universidad San Sebastián, 7510157 Providencia, Santiago Chile; 5 Centro Científico y Tecnológico de Excelencia Ciencia & Vida, Santiago, Chile; 6 Centro de Genómica y Bioinformática, Facultad de Ciencias, Universidad Mayor, Camino La Piramide 5750, 8580000, Huechuraba Santiago, Chile; 7 College of Natural Sciences, Bangor University, Bangor, LL57 2UW UK; 8 Faculty of Health and Life Sciences, Coventry University, Coventry, CV1 5FB UK; 9 Natural History Museum, London, UK; 10 Facultad de Ciencias Biológicas, Universidad Nacional Mayor de San Marcos, Lima, Peru

**Keywords:** Bacterial genetics, Mobile elements

## Abstract

The recent revision of the *Acidithiobacillia* class using genomic taxonomy methods has shown that, in addition to the existence of previously unrecognized genera and species, some species of the class harbor levels of divergence that are congruent with ongoing differentiation processes. In this study, we have performed a subspecies-level analysis of sequenced strains of *Acidithiobacillus ferrooxidans* to prove the existence of distinct sublineages and identify the discriminant genomic/genetic characteristics linked to these sublineages, and to shed light on the processes driving such differentiation. Differences in the genomic relatedness metrics, levels of synteny, gene content, and both integrated and episomal mobile genetic elements (MGE) repertoires support the existence of two subspecies-level taxa within *A. ferrooxidans*. While sublineage 2A harbors a small plasmid related to pTF5, this episomal MGE is absent in sublineage 2B strains. Likewise, clear differences in the occurrence, coverage and conservation of integrated MGEs are apparent between sublineages. Differential MGE-associated gene cargo pertained to the functional categories of energy metabolism, ion transport, cell surface modification, and defense mechanisms. Inferred functional differences have the potential to impact long-term adaptive processes and may underpin the basis of the subspecies-level differentiation uncovered within *A. ferrooxidans*. Genome resequencing of iron- and sulfur-adapted cultures of a selected 2A sublineage strain (CCM 4253) showed that both episomal and large integrated MGEs are conserved over twenty generations in either growth condition. In turn, active insertion sequences profoundly impact short-term adaptive processes. The ISAfe1 element was found to be highly active in sublineage 2A strain CCM 4253. Phenotypic mutations caused by the transposition of ISAfe1 into the *pstC2* encoding phosphate-transport system permease protein were detected in sulfur-adapted cultures and shown to impair growth on ferrous iron upon the switch of electron donor. The phenotypic manifestation of the △*pstC2* mutation, such as a loss of the ability to oxidize ferrous iron, is likely related to the inability of the mutant to secure the phosphorous availability for electron transport-linked phosphorylation coupled to iron oxidation. Depletion of the transpositional △*pstC2* mutation occurred concomitantly with a shortening of the iron-oxidation lag phase at later transfers on a ferrous iron-containing medium. Therefore, the *pstII* operon appears to play an essential role in *A. ferrooxidans* when cells oxidize ferrous iron. Results highlight the influence of insertion sequences and both integrated and episomal mobile genetic elements in the short- and long-term adaptive processes of *A. ferrooxidans* strains under changing growth conditions.

## Introduction

The *Acidithiobacillia* class groups several acidophilic, chemolithoautotrophic γ-proteobacteria that contribute to the geochemical recycling of metals and nutrients in acid-rich environments. Different lines of evidence indicate that there is still considerable unexplored diversity within the class^1–4^. Phylogenetic studies encompassing iron-oxidizing acidithiobacilli have reclassified and reassigned *A. ferrooxidans*-like isolates into several new species^1,5–8^. However, the relatively limited number of available genomic sequences prevents comprehensive comparative genomic studies at both intragenic and intraspecies levels and hinders the discovery and characterization of the true genetic potential of each group. Recently, evidence supporting the existence of subspecies-level taxa within *A. ferrooxidans* species has been recognized^1,2^.

Iron- and sulfur-oxidizers assigned to *A. ferrooxidans *sensu stricto (ATCC 23270^T^; Clade 2)^2,9^ are ubiquitous worldwide and are widely used in bioleaching for the industrial recovery of metals, such as copper and gold^10–12^. Genome sequences of six representative strains of this clade isolated in North and South America (ATCC 23270^T^; ATCC 53993; DSM 16786) and China (DLC5, Hel18; YQH-1) have been publicly released, including two complete chromosomes (ATCC 23270^T^ and ATCC 53993) and one metagenome derived genome (MAG: RVS1)^13–17^. Other genomes from distinct locations have been published in recent years^1^, opening the possibility of further exploring intraspecies diversification and adaptation processes.

Prokaryotes in general, and extremophiles in particular, rely on several sophisticated mechanisms to adapt to extreme environmental conditions and rapidly acclimate to changing conditions. These adjustments entail gene and genome modification mechanisms, among which horizontal gene transfer (HGT) has the greatest impact^18^. HGT is driven by an extensive repertoire of mobile genetic elements (MGEs), including integrative conjugative elements (ICEs), which are self-transferred MGEs. In addition, a few plasmids and a number of discrete integrated MGEs, such as insertion sequences and transposons, have been described in several *A. ferrooxidans* strains^19^. Except for a few MGEs, whose contribution to increased fitness under selective conditions has been demonstrated^20,21^, the adaptive value of MGEs in *A. ferrooxidans* remains mostly unexplored.

To date, at least three genomic islands (GIs) have been described in *A. ferrooxidans* strains^20,22–26^. The first 300-kb GI was shown to be inserted into the tRNA-Ala gene in *A. ferrooxidans* ATCC 23270^T^^26^. This ICE*Afe*1 is integrative^25^, actively excising MGE^23^, and capable of conjugative transfer to suitable recipient strains according to the presence conservation and expression of a complete set of genes encoding self-transfer functions^22^. Several traits relevant to *A. ferrooxidans* physiology are encoded in this element, including gene clusters expressing transfer RNAs^27^, CRISPRs^28^, quorum sensing^29,30^, and exopolysaccharide biosynthesis enzymes^31^. A second 160-kb GI has been discovered in the *rimO* gene of *A. ferrooxidans* ATCC 53993 and contains genes for copper, mercury, and arsenic resistance^20,32^. A third element, partially common to *A. ferrooxidans* ATCC 23270^T^ and ATCC 53993 and designated ICE*Afe*2, has also been identified^24,25,33^ but remains primarily uncharacterized. In addition to these integrated elements, a wealth of insertion sequences (IS), transposases (Tnp), and transposase fragments are scattered in the genomes of the *Acidithiobacillus* spp.^19^. However, their activity has only been demonstrated for some IS elements, including the 1.2-kb ISAfe1 (formerly IST1 or ISTfe1, from the ISL3 family^34^) found in *A. ferrooxidans* and the 1.3-kb ISAfd1 (from the IS701 family) found in *A. ferridurans*, both if which have been associated with a loss of the ability to oxidize/reduce iron^35–38^. Thus, the reversible transposition of mobile genetic elements may be responsible for phenotypic switching in these acidophilic iron/sulfur oxidizers.

In this work, we report an extended comparative genomic analysis of 15 sequenced *A. ferrooxidans* strains and identify sublineages with distinct genomic properties (nucleotide level identity, flexible gene complement, mobile genetic elements pool) that support the existence of subspecies level taxa within the species. The distinction between sublineages is attributable primarily to the acquisitions of mobile genetic elements which carry adaptive gene cargo with the potential to impact long-term adaptive processes. Using strain CCM 4253 as a test case, we further explored aspects of the stability of the different sorts of MGEs and insertion sequences in sublineage 2A during short-term adaptive processes.

## Results and discussion

### Genomic properties of *A*. *ferrooxidans* strains support the existence of two subspecies

**Figure 1:**
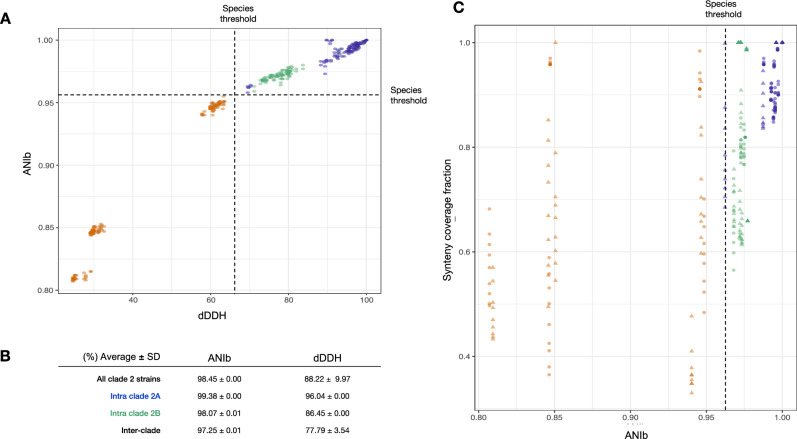
Genomic relatedness and synteny coverage of *A. ferrooxidans* clade 2 strains. (**A**) Average nucleotide identity (ANIb) calculated as in Pritchard et al.^76^ vs. in silico digital DNA–DNA hybridization index (dDDH) assessed using the Genome-to-Genome Distance Calculator with recommended formula 2^77^ and species cut-off limits defined by Meier-Kolthoff et al.^78^ showing a clear-cut distinction between *A. ferrooxidans* clade 2A and 2B strains (excepting strain F221 with comparisons crossing this threshold). (**B**) Basic statistics for intra- and interclade genomic relatedness indexes distributions. Thresholds used for species delimitation are the following: digital DNA:DNA hybridization dDDH > 70% (same genomic species^77^^,^^78^); Average Nucleotide Identity ANI > 96% (same genomic species^73,76^). (**C**) Synteny coverage fraction (using 9 anchors, 2 to 10 calculated as in Drillon et al.^59^) between *A. ferrooxidans* strains sublineage 2A and 2B showing high levels of synteny between strains of the same sublineage (in blue; 2A vs. 2A or 2B vs. 2B strains) with respect to inter sublineage comparisons (in green; 2A vs. 2B and 2B vs. 2A strains). Strain ATCC 23270^T^ (circles) is a reference strain for the 2A sublineage and strain PQ505 (triangles) for the clade 2B sublineage. Interspecies comparisons in brown are included as a control (*A. ferrianus* DSM 107098*, A. ferridurans* ATCC 33020*, A. ferriphilus* DSM 100412, and *`A. ferruginosus´* CF3).

The sequenced *A. ferrooxidans* strains used in this study have comparable genomic features in size, GC content, and global coding potential (Supplementary Table 1). Although all strains in this clade possess two rRNA operons with identical gene contexts^2^, they differ in the number of tRNAs (Supplementary Table 1). In the case of the ATCC 23270^T^ strain, this is due to the presence of an additional tRNA set in a foreign MGE known as ICE*Afe*1^23,27^. However, the origin of the additional tRNAs in other strains is currently undetermined (e.g. DSM 16786). All these strains have nearly identical (> 99.2%) 16S rRNA genes, are highly conserved genome-wise, with an average nucleotide identity (ANIb) ranging between 95.9 and 100%, and in silico DDH values averaging 88.2% (Supplementary Table 2). Hence, they can be unambiguously assigned to a single species of *A. ferrooxidans*. Despite this fact, two well-defined subgroups of strains (sublineages 2A and 2B) are evident from both genomic indexes calculated (Fig. 1A), with reciprocal ANI and DDH average values much closer to the species delimitation thresholds than the group-specific averages, suggesting the existence of two subspecies (Fig. 1B).

Since gene order conservation (synteny) is lost faster than sequence similarity^39^, we compared the synteny levels among *A. ferrooxidans* strains belonging to both 2A and 2B sublineages to assess the existence of subspecies. Using distinct iron-oxidizing *Acidithiobacillus* species as controls (47–92% synteny coverage), intermediate synteny coverage levels were observed within *A. ferrooxidans* strains when sublineages were disregarded (Fig. 1C, green; 2A vs. 2B and 2B vs. 2A strains). On the contrary, cross-comparison of strains of the same sublineage confirmed high synteny levels (Fig. 1B, blue; 2A vs. 2A or 2B vs. 2B strains). Accordingly, strains BY03, PQ505, and PQ506 are recognized here as subspecies *A. ferrooxidans* subsp. *andinus* PQ505^T^ (sublineage 2B) while *A. ferrooxidans* subsp. *ferrooxidans* ATCC 23270^T^ is retained for sublineage 2A strains. Strain F221 could represent an additional sublineage, yet the evidence gathered so far is not conclusive.

### Pangenome analysis reveals substantial differences in the coding potential of the subspecies

**Figure 2:**
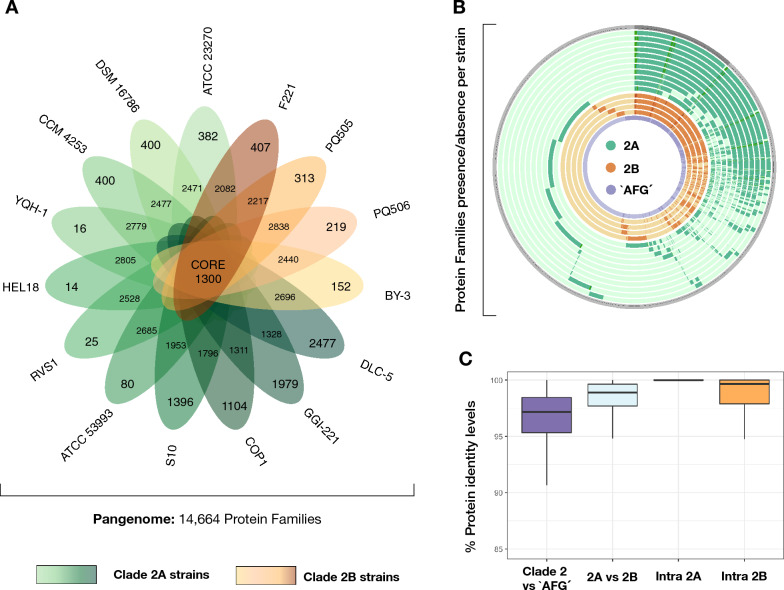
Pangenome analysis of *A. ferrooxidans* strains pertaining to sublineages 2A and 2B. (**A**) Quantitative (Venn diagram) and (**B**) qualitative (Circos plot) comparative analysis between 2A (shaded in green) and 2B (shaded in orange) sublineage strains showing the core and flexible and exclusive protein families derived using GET_homologues^79^ as described in Moya-Beltrán et al.^1^. (**C**) Percentual protein identity levels between conserved proteins occurring in all species strains (2A versus 2B) or within sublineages (2A or 2B). *A. ferrooxidans*-closely related species ‘*A. ferruginosus* CF3’^1^ was included in the comparison as inter-species control (shaded in purple).

We next analyzed the coding potential of 15 publicly available complete and draft genomes of *A. ferrooxidans* and derived the core, flexible, and exclusive gene complements for this set of strains. The core genome of the species, composed of protein-coding gene sequences common to all 15 strains, consisted of 1,300 protein families (Fig. 2A Supplementary Table 3A). This set represents 42% of the CDSs encoded by *A. ferrooxidans* ATCC 23270^T^ and 9% of the total pangenomic gene complement of the species. Comparative analysis of this set of core proteins between strains assigned to each of the sublineages (Fig. 2B) revealed higher percentages of conserved proteins and higher average protein identity/similarity levels at the sublineage level (Fig. 2C; Supplementary Table 3B), confirming the results obtained for the group at the nucleotide level. While 64 protein families present in the majority (> 90%) of the sublineage 2A strains are exclusive to this group, only 30 protein families are exclusive to the sublineage 2B strains (Supplementary Table 3C). This contrasts the 427 protein families exclusive to ‘*Acidithiobacillus ferruginosus*’ CF3^1^ compared to *A. ferrooxidans* strains, which underly species differentiation in terms of gene content. Functional assignments of the flexible and exclusive gene complements are listed in Supplementary Table 3D and are further analyzed below. Apart from hypothetical or unknown function proteins, which comprised 79% of the flexible and exclusive gene complement of all *A. ferrooxidans* strains compared, the most frequent functional categories in the exclusive gene complement of sublineages 2A and 2B were addiction module proteins and pili-related functions. To learn more about the nature and organization of the dispensable/flexible genome and its contribution to subspecies differentiation, we predicted and analyzed the repertoire of MGEs in *A. ferrooxidans* strains.

### *A*.* ferrooxidans* subspecies can be distinguished by their integrative MGEs repertoire

An integrated approach employing existing MGE prediction and pangenome analysis tools was designed to identify MGEs in the genomes of sequenced *A. ferrooxidans* strains belonging to the sublineages 2A and 2B, several of which remain as draft genomes (Supplementary Fig. 1). The strategy used aims to bypass the fragmentation of most drafts (steps 1–3), the poor annotation of MGE-related contigs (steps 4–6), and the difficulties of fully reconstructing the elements present in the dataset (step 7). For this purpose, we used information available on several integrated MGEs (iMGEs) that have been identified and described to a different extent in *A. ferrooxidans*^16,20,22,23,27,33^. Moreover, candidate MGEs derived from applying existing MGE prediction resources to two available complete *A. ferrooxidans* genome sequences (NC_011761, NC_011206) were used^33,40^.

**Figure 3:**
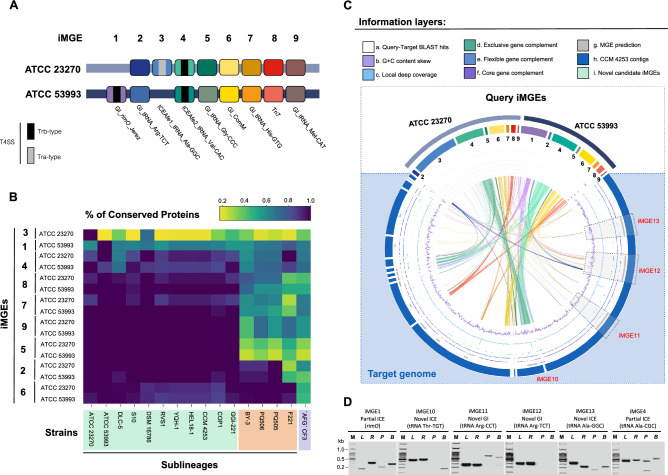
Location, occurrence, and mapping of MGE-associated genes from validated and candidate iMGEs in the draft genome of *A. ferrooxidans* CCM 4253. (**A**) Location of experimentally validated and bioinformatically predicted iMGEs (candidate) present in the two query genomes and their correlative order (iMGE1 to iMGE9). Query iMGEs were classified as Tra- or Trb-type Integrative Conjugative Elements (ICEs), Genomic Islands (GIs), or Tn7 transposons (Tnp) and their integration sites were identified (detailed in Table 1). (**B**) Occurrence and coverage of validated and candidate iMGEs of *A. ferrooxidans* ATCC 23270^T^ and ATCC 53993 in an extended set of draft genomes of the species. Strains were clustered based on the coverage patterns of the MGE-associated gene products (cut-off: e-value -10; detailed in Supplementary Table 4). (**C**) Identification of novel iMGEs and iMGE-fragments in strain CCM 4253. Colored elements in the outer layer are known and candidate iMGEs in ATCC 23270^T^ and ATCC 53993 genomes. Some iMGEs are strain-exclusive, such as iMGE1^20^ and iMGE3^23^. Others are conserved in two reference strains used as queries. Dark blue elements correspond to CCM 4253 strain genomic assembly contigs (GCA_003233765). Additional information layers, from the center of the figure outwards, correspond to (a) the TBlastN hits found in the CCM 4253 genome using either query strains MGEs; (b) the G + C content skew of the position; (c) the deep local coverage at the position; (d–f) the exclusive, flexible, or core pangenome compartment to which a given CCM 4253 gene pertains and (g) the prediction of MGE features using a combination of tools as previously described in Gonzalez et al.^40^ and Moya-Beltrán et al.^33^. (**D**) PCR validation of novel and partially conserved candidate iMGEs identified in the genome of strain CCM 4253. PCR products correspond to specific *attL*, *attR*, *attP*, and *attB* sites (the scheme of experimental design is shown in Supplementary Table 4D and primer sequences are listed in Supplementary Table 4E) of the following iMGEs: iMGE1 (partial ICE integrated into the *rimO* gene); iMGE10 (integrated at tRNA Thr-TGT); iMGE11 (integrated at tRNA Arg-CCT); iMGE12 (integrated at tRNA Arg-TCT); iMGE13 (integrated at tRNA Ala-GGC); iMGE4 (partial ICE integrated at tRNA Ala-CGC). PCR of *attP* and *attB* sites were evaluated on DNA recovered after incubation of *A. ferrooxidans* CCM 4253 cells with mitomycin C. Lane M represents the 100 bp DNA ladder; lanes L, R, P, and B represent the *attL*, *attR*, *attP*, and *attB* specific sites in the individual iMGEs, respectively. The gel image has been cropped for display. The original gel image is shown in Supplementary Table 4F.

A total of nine iMGEs spanning experimentally validated and bioinformatically predicted GIs, ICEs, or Tn7 transposons were present in the query genomes pertaining to *A. ferrooxidans* strains ATCC 23270^T^ and ATCC 53993 (Table 1). These were numbered correlatively (iMGE1–9) according to their position along the genome alignment of both strains (Fig. 3A). Protein-coding genes of all iMGEs were used as queries to assess the occurrence and percentage coverage of each iMGE in other *A. ferrooxidans* strains (Fig. 3B, Supplementary Table 4A). The orthology of the queried iMGE-associated proteins and candidate target proteins in the draft genomes of the other strains was confirmed by reciprocal best hits with BLAST (data not shown). This strategy resulted in a total of 10,604 target protein hits above a set cut-off (e-value < 0.001), which were distributed among 15 strains, including two query genomes for which iMGEs have been detected but not yet characterized^16,41^. The heatmap in Fig. 3B shows that sublineage 2A iMGEs are poorly conserved in *A. ferrooxidans* sublineage 2B strains, except for iMGE6 and iMGE2, which are present and conserved in most strains of this species, suggesting early fixation in the evolutionary history of the clade. Identified target proteins were analyzed for their likelihood of iMGE affiliation by analyzing contextual information (Supplementary Fig. 1; step 5), including G + C content skew, depth coverage, an affiliation of flanking proteins to the exclusive, flexible, or core gene complement of the species or to regions classified as foreign or mobile by publicly available software.

**Table 1:** Features of iMGEs identified in sequenced *A. ferrooxidans* strains.

	MGE_ID	Type MGE	Status	General features				Att sites		Functional modules				Accessory modules	Occurrence*
				Locus	Size (Kb)	#CDS	G + C%	attB	attL/R	Int/Tnp genes	T4SS	TA system	Partition system		Strains
1	iMGE1_ATCC53993	GI	V^b^	119749..285884	165	187	57.2	*rimO*	DR(*rimO*,–)	–	Trb	Doc	ParAB	Type_I RM, Mercury_resistance_operon	ATCC 53,993
2	iMGE2_ATCC23270	GI	C	748759..770164	21.4	21	57.2	Arg-TCT	DR(77,48)	RitABC	–	–	–	Type I RM	ATCC 23,270, ATCC 53,993, GGI-221, S10, DSM 6786, RVS1, YQH-1, Hel18, CCM4253, COP1, DLC-5, PQ505, PQ506, BY-3
3	iMGE3_ATCC23270	ICE	V^a^	909149..1200493	29.1	364	57.1	Ala-GGC	DR(76,48)	–	Tra	VapB-VapC, MazE-MazF	–	CRISPR-Cas Type IV system	ATCC 23,270
4	iMGE4_ATCC23270	ICE	C	1346955..1522548	17.6	197	57.2	Val-CAC	DR(75,49)	Int	Trb	YafQ, Phd-Doc, HigA	ParAB	Type I RM	ATCC 23,270, GGI-221, S10
5	iMGE5_ATCC23270	GI	C	2117161..2131886	14.7	13	58.7	Gly-CCC	DR(71,–)	–	–	–	–	Type I RM	ATCC 23,270, ATCC 53,993, GGI-221, S10, DSM 6786, RVS1, YQH-1, Hel18, CCM4253, COP1, DLC-5
6	iMGE6_ATCC23270	GI	C	2137678..2232582	94.9	117	56.4	*comM* Integrase	DR(702,14)	Int, RitABC		–	–	Res	ATCC 23,270, ATCC 53,993, GGI-221, S10, DLC-5, PQ505, PQ506, BY-3, F221
7	iMGE7_ATCC23270	GI	C	2462004..2478265	16.2	22	53.4	His-GTG	DR(76,49)	–	–	VapB	–	PilT	ATCC 23,270, ATCC 53,993, GGI-221, S10, DSM 6786, RVS1, YQH-1, Hel18, CCM4253, COP1, DLC-5
8	iMGE8_ATCC23270	GI	C	2854800..2882600	27.8	33	52.2	Tn7	DR(–,–)	Tn5468 Tnp	–	–	–	Type III RM, Mrr	ATCC 23,270, ATCC 53,993, GGI-221, S10, DSM 6786, RVS1, YQH-1, Hel18, CCM4253, COP1, DLC-5
9	iMGE9_ATCC23270	GI	C	2944843..2965000	20.2	24	61	Met-CAT	DR(–,–)	ISAfe3 Tnp (us)	–	–	–	[Ni/Fe] hydrogenase	ATCC 23,270, ATCC 53,993, GGI-221, S10, DSM 6786, RVS1, YQH-1, Hel18, CCM4253, COP1, DLC-5

*iMGE* integrated mobile genetic element, *GI* genomic island, *ICE* integrated conjugative element, *C* candidate, *V* validate; *comM* gene encoding competence, *Tn7* transposon Tn7, *rimO* gene encoding ribosomal protein S12, *DR* direct repeat (total gene, total repeat) on bp, *Int* integrase, *Tnp* transposase, *Us* upstream.

(*) Occurrence ≥ 95% according to Supplementary Table 4A and the reference genome.

^a^Bustamante et al.^23^. ^b^Orellana et al.^20^.

Integrating these pieces of information, new candidate MGEs (or MGE fragments) were identified (Supplementary Table 4A). For illustrative purposes, we chose strain CCM 4253 as a test case based on the quality and contiguity of its draft genome (Fig. 3C). The main characteristics of the novel candidate iMGEs identified in strain CCM 4253 are summarized in Table 2 (see Supplementary Table 4C for further details). These include two novel ICEs (iMGE10 and iMGE13), two novel GIs (iMGE11 and iMGE12), and two variant versions of the ATCC 23270^T^ iMGE1 and iMGE4. The occurrence of novel iMGEs at the predicted integration sites in the genome of strain CCM 4253 was experimentally validated for all six of these iMGEs, while mitomycin C-inducible excision could be demonstrated in five of them (Fig. 3D, the primer sequences are listed in Supplementary Table 4D).

**Table 2:** Features of novel iMGEs identified in *A. ferrooxidans* CCM 4253.

	iMGE ID	iMGE Type	# of segments	General features				Insertion site	Functional modules				Accessory modules	Occurrence*
				Location	Size (Kb)	# CDS	G + C%	*attB*	Int genes	Trb-type T4SS	MOB + CP	Partition system		Strains
1	iMGE1_CCM4253 (Partially conserved)	ICE	68	QKQP01000006 195,438..248187	53	60	59.74	*rimO*	–	VirB1, VirB3, VirB4, VirB5, VirB6, VirB8, VirB9, VirB10, VirB11	VirD2: MOBP_A VirD4: t4cp2	ParAB	Hypotheticals	CCM 4253, YQH-1
2	iMGE4_CCM4253 (Partially conserved)	ICE	57	QKQP01000011 1..14511 QKQP01000002 1..121640	136	144	56.4	Ala-CGC	Int	VirB3, VirB4, VirB5, VirB6, VirB8, VirB9, VirB10, VirB11	VirD2: MOBP_A VirD4: t4cp2	ParAB	Rubisco, carboxysome	CCM 4253
3	iMGE10_CCM4253 **Novel**	ICE	44,45	QKQP01000001 975,082..1056184	81	95	57.96	Thr-TGT	Int	VirB1, VirB3, VirB4, VirB6, VirB8, VirB9, VirB11,	VirD2: MOBP_A VirD4: t4cp2	ParB	Type I restriction-modification system	CCM 4253, YQH-1
4	iMGE11_CCM4253 **Novel**	GI	44	QKQP01000001 935,930..972728	37	34	56.21	Arg-CCT	Int	–	–	–	Diguanylate cyclase/phosphodiesterase with PAS/PAC sensors	CCM 4253
5	iMGE12_CCM4253 **Novel**	GI	39,40	QKQP01000001 537,863..705907	168	166	56.44	Arg-TCT	Int	–	–	–	CusABC complex, CzcABC complex, *merRTPC* genes Type II restriction-modification system	CCM 4253
6	iMGE13_CCM4253 **Novel**	ICE	37	QKQP01000001 282,464..397058	115	125	57.88	Ala-GGC	Int	VirB1, VirB2, VirB3, VirB4, VirB5, VirB6, VirB8, VirB9, VirB10, VirB11	VirD2: MOBP_A VirD4: t4cp2	–	Cellulose synthase	CCM 4253, YQH-1, Hel18

*iMGE* integrated mobile genetic element, *GI* genomic island, *ICE* integrated conjugative element, *rimO* gene encoding ribosomal protein S12, *Int* integrase.

(*) Occurrence ≥ 95% according to Supplementary Table 4C.

### *A*.* ferrooxidans* subspecies 2A harbors episomal MGEs absent in 2B strains

**Figure 4:**
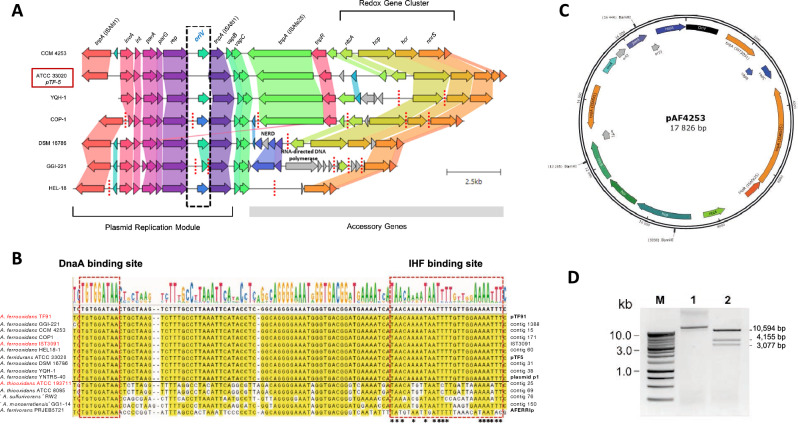
Analysis of candidate eMGEs in the genomes of *A. ferrooxidans* strains. (**A**) Stand-alone contigs and contig fragments aligned against *A. ferridurans* ATCC 33020^T^ plasmid pTF5 (NC_005023) showing modular organization and variations among sublineage 2A strains. (**B**) The alignment of the *oriV* region identified in the replication module of pTF5-like plasmids of *A. ferrooxidans* sublineage 2A strains and matching sequenced recovered from NCBI. (**C**) A schematic map of the pTF5-like plasmid of strain CCM 4253 showing contig circularization (17,826 bp). The localization and orientation of predicted genes and the position of the putative origin of replication are indicated. (**D**) *Bam*HI restriction analysis of pTF5-like plasmid of strain CCM 4253. Lane M represents a 1-kb DNA ladder; lane 1 represents uncut plasmid DNA; lane 2 represents plasmid DNA digested with a BamHI-HF® restriction endonuclease (New England Biolabs) for 1 h at 37 °C. The plasmid and restriction products were analyzed by electrophoresis in a 0.7% agarose gel. The gel image has been cropped for display. The original gel image is shown in Supplementary Table 5B. Accession numbers for the *A. ferrooxidans* genomic sequences in section B are listed in Supplementary Table 1B, and additional sequences in the alignment are the following: YNTRS-40 p1 (NZ_CP040512.1); ATCC 19377 (AFOH01000025); ATCC 8085 (JABBDT010000069), RW2 (JAAOMP010000076); GG1-14 (JABBOU010000150); AFERRIp (NZ_LT841306.1); pTF91 (U14129); AFE GGI-221 (AEFB01001388); AFE CCM 4253 (QKQP01000015); AFE COP1 (JABBDN000000171); AFE IST3091 (U32113.1); AFE HEL18 (LQRJ01000060); AFE DSM 16786 (JABFOH000000031); AFE YQH-1 (LJBT01000038); AFE TNTRS-40, plasmid p1 (CP040512.1); ATH ATCC 19377 (AFOH01000025); ATH ATCC 8085 (JABBDT010000069); ‘ASU’ RW2 (JAAOMP010000076); ‘AMO’ GG1-14 (JABBOU010000150); AFV PRJEB5721, AFERRIp (LT841306.1); AFD ATCC 33020 (NC_005023). Abbreviations of species names are the following: AFE, *A. ferrooxidans*; AFD, *A. ferridurans*; ATH, *A. thiooxidans*; ASU, ‘*A. sulfurivorans*’; AMO, ‘*A. monserratiensis*’; AFV, *A. ferrivorans*.

The MGE prediction pipeline used in this work produced several hits in stand-alone contigs that failed to incorporate into the chromosome scaffolds or the circular assemblies of the draft genomes. Some exhibited genomic signatures of plasmids and were highly conserved exclusively among 2A strains (Fig. 4). BLASTn analysis of these contig segments against the non-redundant NCBI database revealed a high level of sequence identity (> 88%) and synteny coverage against a member of the pTFI91-like plasmid family, specifically *A. ferridurans* ATCC 33020^T^ plasmid pTF5^42^. This plasmid family has initially been described in *A. ferrooxidans* strains^43^ and found in other *Acidithiobacillus* spp.^42^. They share a conserved 2.2-kb *Sac*I restriction endonuclease region containing the replication origin (*oriV*). The *oriV* region of *A. ferrooxidans* 2A candidate plasmids identified downstream of the *rep* was found to be part of a gene cluster encoding invertase, integrase, partition genes *parA* and *parG,* and plasmid replicase, flanked by two ISAfd1-like transposases, likely forming a distinct insertion sequence (Fig. 4A). The identified *oriV* is highly conserved compared to that described for pTF5. It exhibits binding sites for DnaA and IHF family proteins (Fig. 4B), which supports the assignment of these contigs as episomal MGEs (eMGEs). The existence of a pTF5-like plasmid was confirmed in silico in *A. ferrooxidans* CCM 4253 by recirculation of the contig 15 (QKQP01000015) (Fig. 4C) and then experimentally by isolation and cleavage of the replicon using the restriction endonuclease BamHI, resulting in three predicted products of 3,077, 4,155, and 10,594 bp (Fig. 4D). In addition to the replication module, the pTF5-like plasmids identified in *A. ferrooxidans* 2A strains encoded (i) a single addiction or *vapBC*-type module, (ii) an adaptation module comprising genes encoding redox-active proteins predicted to function in electron transport systems (*ntcA / fnr*, *hcp*, *hcr*, *nnrS*), and (iii) an ISAfe25 transposon consisting of *tnpARX* genes. The ISAfe25 transposon is replaced in two strains by a cluster of three genes encoding retron-type RNA-directed DNA polymerase, StbC protein, and putative NERD domain protein. The gene cluster encoding the redox-active proteins forms the essential part of the sequence of the pTF5-like plasmids, which distinguishes these larger plasmids from the 9.8-kb pTFI91 plasmid^43^. The adaptation genes *ntcA*, *hcp,* and *hcr* (with 99% identity) were also part of the flexible gene complement of plasmid-free *A. ferrooxidans* strains, including the ATCC 23270^T^ (ICE*Afe*2) and sublineage 2B strains (near MGE-signature genes such as *trb*-type T4SS genes, data not shown). Complete annotation of the pTF5-like plasmid from strain CCM 4253 is provided in Supplementary Table 5 and referred to hereafter as pAFE4253.

### Flexible gene modules of the *A*. *ferrooxidans* mobilome reveal the adaptive value

**Figure 5:**
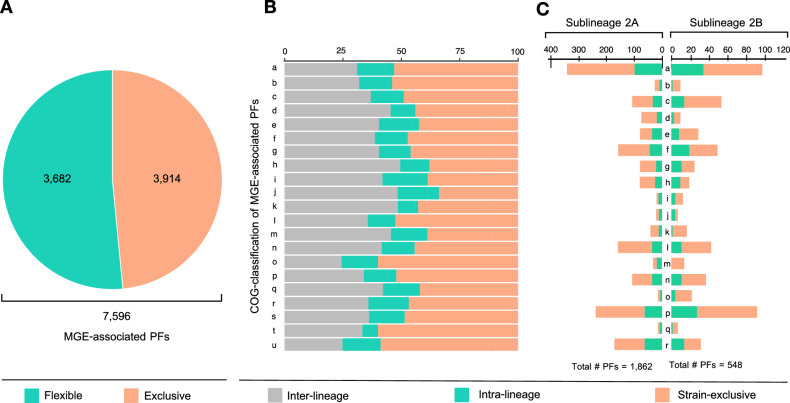
Functional potential of MGE-associated genes in *A. ferrooxidans* strains of sublineages 2A and 2B. (**A**) The abundance of exclusive (present in a single strain of the set under comparison, organge) and partially shared or flexible (present in less than 95% of the strains in the set, green) MGE-associated genes, regardless of the strains' sublineage. (**B**) COGs functional gene classifications of exclusive and flexible MGE-associated genes and their relative abundance (%). Color-coding according to the gene occurrence in single strains of either sublineage (exclusive, orange), strains from the same sublineage (intra-lineage, green), or several strains from both sublineages (inter-lineage, grey). (**C**) Sublineage-enriched COGs functional gene categories. COG categories are as follows: (a) RNA processing and modification; (b) chromatin structure and dynamics; (c) energy production and conversion; (d) cell cycle control, cell division, and chromosome partitioning; (e) amino acid metabolism and transport; (f) nucleotide transport and metabolism; (g) carbohydrate transport and metabolism; (h) coenzyme transport and metabolism; (i) lipid transport and metabolism; (j) translation, ribosomal structure, and biogenesis; (k) transcription; (l) replication, recombination, and repair; (m) cell wall/membrane/envelope biogenesis; (n) cell motility; (o) posttranslational modification, protein turnover, chaperones; (p) inorganic ion transport and metabolism; (q) secondary metabolites biosynthesis, transport, and catabolism; (r) general function prediction only; (s) function unknown; (t) signal transduction mechanisms; and (u) intracellular trafficking, secretion, and vesicular transport (https://ftp.ncbi.nih.gov/pub/COG/COG2020/data/fun-20.tab). Total number of exclusive and flexible MGE-associated protein families with COGs assignment (categories a to r) is indicated.

To assess the functional diversity of the *A. ferrooxidans* mobilome and the contribution of its gene cargo to sublineage differentiation, the gene neighborhoods of MGE-associated proteins (queries and targets) derived from the *A. ferrooxidans* closed and draft genomes were further explored. Ten genes in the vicinity of seeds, or seed clusters (or all genes of smaller contigs from draft genomes), were recovered from both DNA strands. Boundaries between the MGEs and the chromosome were inferred by assigning the individual protein products to the core, flexible, or exclusive pangenome gene complements. Only genes pertaining to the flexible or exclusive gene pool were retained for further analysis (Supplementary Table 6). This strategy produced 7596 protein families associated with known and novel MGEs of the species and its sublineages (Fig. 5A). As a result, 51.8% of the *A. ferrooxidans* pangenome is tentatively associated with the mobilome.

Functional assignment of these genes, and functional classifications achieved using CDD, COGs, and KEGG revealed that 5.7% of MGE-associated protein families encode functions related to MGE biology (replication, recombination/integration, mobilization/conjugation, maintenance/stability, partition), 5.9% encode accessory functions with potential adaptive value (defense systems, transporters, enzymes), and 16.6% lack yet functional assignment (Supplementary Table 6). Representative results of the protein family (PF) distributions per functional class obtained using COGs are shown in Fig. 5B. COG enrichment analysis showed that a subset of 137 protein families (comprising 5063 individual proteins) were exclusively assigned to single strains of one sublineage (Fig. 5B, exclusive PFs in orange) or to several strains from the same sublineage (Fig. 5B, intra-lineage PFs in green). As expected from the number of strains in each dataset (11 strains in clade 2A vs 4 strains in clade 2B), the number of protein families per COG category indicated that the flexible genome of the sublineage 2A is larger than that of sublineage 2B (Fig. 5C) and supports the view that the pangenome of both sublineages is open. Integrated functional classification of the subset of MGE-associated protein families using COGs, CDD and KEGG revealed that both sublineages have a distinct set of glycosyltransferases and restriction-modification systems. In contrast, strains of both sublineages differ in the number and types of transporters, transposases, and transcriptional regulators, which are invariantly more abundant and diverse in the non-type strains (Supplementary Table 6).

### ISs affect the electron donor adaptation of sublineage 2A strain CCM 4253

To evaluate the persistence and/or mobility of iMGEs and eMGEs in *A. ferrooxidans* strains under different growth conditions, we chose sublineage 2A strain CCM 4253 as a test case. In iron-oxidizing acidithiobacilli, changes in growth and stress conditions have proved to increase the transposition of IS elements at new sites, particularly in genes involved in iron oxidoreduction^36–38^. Thus, we performed genome resequencing of long-term iron- and sulfur-adapted cultures (*Experiment 1*, 20 generations). We also monitored genotype and phenotype over time upon switching culture media in focused short-term adaptations (*Experiments 2 and 3*, 6 generations; Supplementary Fig. 2).

**Figure 6:**
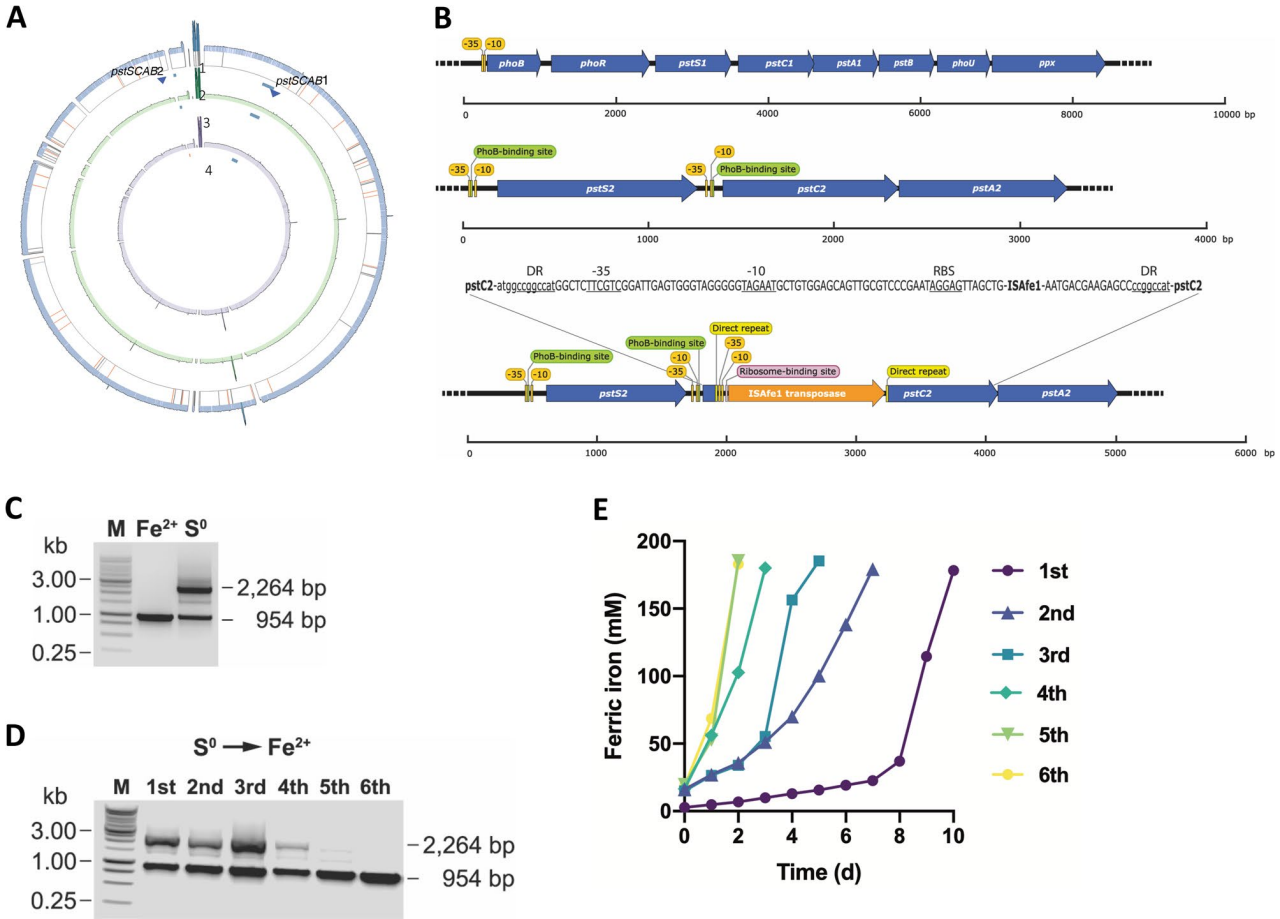
Influence of ISs in the long-term and short-term adaptation of 2A sublineage strain CCM 4253 to an energy substrate switch. (**A**) Ordered CCM 4253 pseudochromosome and genome resequencing Circos maps showing replicative transposition events (1) occurring during the growth of iron- (green) and sulfur-adapted (purple) cultures. Concentric lanes represent the reads coverage of (2) original sequencing, (3) iron-grown cultures resequencing, technical replicates, and (4) sulfur-grown resequencing cultures, technical replicates. ISs (grey, Supplementary Table 5A), except ISAfe1, highlighted in orange. Supporting alignments in bam format and coverage values are provided in Supplementary Table 5C and Figshare (https://doi.org/10.6084/m9.figshare.20523591). (**B**) Standard organization of the *pstI* and *pstII* operons and mutant ∆*pstII* operon in the *A. ferrooxidans* CCM 4253 chromosome. The position of the ISAfe1 element inserted into the open reading frame of the *pstC2* gene is indicated. Putative promoter regions (− 10 and − 35), PhoB-binding sites, ribosome binding site (RBS), and direct repeats (DR) are indicated. Lowercase letters indicate the sequence of the *pstC2* gene, while uppercase letters indicate the sequence of the IS element. The scales under each operon indicate the sequence size (bp). (**C**) PCR evaluation of the transpositional mutation in the long-term iron- and sulfur-adapted cultures, as shown in Supplementary Fig. 2 (*Experiment 1*). Lane M represents a 1-kb DNA ladder; lane Fe^2+^ represents the PCR product of the *pstC2* locus from the iron-adapted culture; lane S^0^ represents the PCR product of the *pstC2* locus from the sulfur-adapted culture. The gel image has been cropped for display. The original gel image is shown in Supplementary Fig. 2D. (**D**) PCR evaluation of reversal of the transpositional mutation during subsequent culture transfers, as shown in Supplementary Fig. 2 (*Experiment 3*). Lane M represents a 1-kb DNA ladder; lanes 1–6 represent the PCR product of the *pstC2* locus from the first to sixth individual iron passages in Fig. 6E. The gel image has been cropped for display. The original gel image is shown in Supplementary Fig. 2E. (**E**) The long-term sulfur-adapted *A. ferrooxidans* CCM 4253 mutant strain Δ*pstC2* passaged on ferrous iron repeatedly, as shown in Supplementary Fig. 2 (*Experiment 3*). The first (purple circle), second (dark blue triangle), third (light blue square), fourth (green diamond), fifth (light green inverted triangle), and sixth (yellow circle) iron passage.

Genome resequencing of iron- and sulfur-adapted cultures of strain CCM 4253 (*Experiment 1*) did not show any significant changes in the location of iMGEs with respect to genome reference (QKQP01), which occurred at the exact inferred locations in both derived cultures (Fig. 6A). Plasmid pTF5-like (QKQP01000015) was stably retained by cells from both adapted cultures and occurred at comparable fold proportions (sixfold: Iron; fivefold: Sulfur; Supplementary Table 5C). Instead, the reconstructed chromosomes of the iron- and sulfur-adapted cell populations differed by a single replicative transposition of the ISAfe1 in the sulfur-adapted culture (Fig. 6A,B). The strain CCM 4253 carries 12 distinct ISs and 28 copies of the ISAfe1 (ISL3) spread along its genome (Supplementary Table 7). In the sulfur-adapted culture, an additional copy of the ISAfe1 interrupted the *pstC2* gene (DN052_16065) encoding an identical phosphate permease (AFE_1940, 100% identity) to that found in the *A. ferrooxidans* ATCC 23270^T^ genome^17^. The *A. ferrooxidans* type strain's genome contains two similar *pstC* genes, but their amino acid sequence shares only 29% identity. All genomes analyzed in this work encoded two *pst* operons that contribute to Pi uptake: (i) *pstI* operon with sensor and regulatory functions and the exopolyphosphatase encoding gene (*phoBR-pstS1C1A1B1-phoU-ppx*), and (ii) stand-alone partially incomplete transporter *pstII* operon (*pstS2C2A2*) (Fig. 6B). Both permeases form part of the binding-protein-dependent transport systems for inorganic phosphate (Pi), the Pst systems found in many bacterial species. The phosphate transporter PstSCAB activates the histidine kinase PhoR under Pi-limiting conditions, which subsequently phosphorylates the transcription factor PhoB and thus activates the *pho* regulon, allowing Pi uptake. In contrast, PhoB is deactivated by PhoR under sufficient Pi conditions, consequently inhibiting the expression of genes involved in response to Pi starvation^44^. The expression of both *pstI* and *pstII* operons is induced in *A. ferrooxidans* ATCC 23270T under phosphate limitation in modified 9 K-Fe media at pH 1.5 (personal communication, Mario Vera). Also, the expression of the *pstII* operon is higher (sixfold) in 9 K phosphate rich media when grown in iron at pH 1.8 compared to elemental sulfur at pH 3.5, while the *pstI* operon is equally expressed in both conditions (personal communication, David S. Holmes). This evidence suggests that the *pstII* operon is preferentially used for phosphate uptake in low pH iron media (where iron oxidation may cause phosphate depletion via chelation or precipitation), and that the inactivation of this transporter may occur and be selected for when cells are grown in the absence of iron and/or at higher pH. Although the ISAfe1 element silenced the *pstC2* gene related to phosphate metabolism, the same element has previously been shown to silence the *resB* gene with a completely different function. The *resB* gene encodes the maturation protein required to form the *bc*_1_ complex involved in reverse electron transfer during iron oxidation in *A. ferrooxidans*. An interruption of the *resB* gene in strain ATCC 19859 resulted in a mutant that lost the capacity to oxidize iron but retained the ability to oxidize sulfur^37^. Furthermore, the stress induced by elevated sodium chloride concentration caused ISAfd1 to be inserted downstream of the two promoters PI and PII of the *rus* operon (which encodes the iron oxidation pathway), thereby preventing its transcription. The ability to oxidize iron was restored after prolonged cultivation in the absence of sodium chloride, and two revertant strains were obtained^38^. Given the scarcity of genetic mutants of the taxon, we analyzed this mutation in further detail.

### Mutational inactivation of *ptsC*2 in sulfur-grown cells impairs early iron oxidation

In contrast to previously described transposition mutations in iron-oxidizing acidithiobacilli, the sulfur-adapted *A. ferrooxidans* culture with △*pstC2* mutation retained the ability to oxidize iron but only after a lag phase lasting several days. A similarly long lag phase has been observed in *A. ferrooxidans* cells after switching e^-^ donor from sulfur to iron and not vice versa^45^. This lag period is considered to reflect the time required to synthesize regulatory factors to induce genes involved in iron oxidation^46,47^. Although cell adaptation during the sulfur-to-iron transition has been comprehensively described at the mRNA and protein levels, regulatory factors responsible for this lag phase remain unidentified^48^. Further investigation, using PCR-based screening of *pstC2* alleles (see Methods and Supplementary Table 4D) revealed that the long-term sulfur-adapted culture contained not only the △*pstC2* transposition mutant (product size of 2264 bp) but also the *pstC2* wild-type allele (product size of 954 bp) (Fig. 6C). Thus, we sought to analyze the consequences of this mutation.

We monitored the culture transfer in which the transposition event occurred or disappeared. When transferring a long-term iron-adapted culture (pH 1.7) that is wild type for both *pstC1* and *pstC2* alleles to sulfur medium (initial pH 3.5), and then repeatedly transferring a 10% inoculum to fresh sulfur medium (*Experiment 2*), a PCR product of 2264 bp indicating the emergence of the △*pstC2* allele was observed from the sixth generation onwards. Although the same mutation was repeatedly observed, a PCR product of 954 bp corresponding to the *pstC2* allele was still detected in the culture. In addition, the sulfur-adapted populations enriched in the △*pstC2* mutant were repeatedly observed to stop growing on elemental sulfur when the pH reached pH 1.8 (instead of pH < 1.3 as was the case of wild type cultures), implying the mutants are probably sensitive to pH. This implies that the populations enriched in cells bearing the mutant allele are probably exposed to severe acidification leading to culture collapse. In turn, the reciprocal experiment (*Experiment 3*), entailing the repeated transfer of a long-term sulfur-adapted culture in a fresh iron-containing medium stably maintained at pH 1.7, revealed the coexistence of both the *pstC2* and △*pstC2* alleles until the fourth generation, after which the transposition event stopped being detected (Fig. 6D). In parallel the iron oxidation lag phase shortened gradually with each transfer to fresh iron media until it disappeared entirely after the fourth transfer (Fig. 6E), a point at which cultures were well adapted to growth in iron.

These results indicate that sulfur-adapted cultures growing under higher pH endure or select the emergence of the △*pstC2* allele. Phosphoric acid has three *p*K_a_ values, the lowest of which is 2.1. Only anionic dihydrogen phosphate and undissociated phosphoric acid are relevant in extremely acidic environments. The latter becomes increasingly dominant with decreasing pH (as occurs with sulfur oxidation, not iron oxidation). Thus, an enhanced influx of dihydrogen phosphate and phosphoric acid into the cells expressing both phosphate transporters may lead to cell death by acidification of the cytoplasm and ultimately to culture collapse. Also, our results suggest that iron-adapted cultures growing at lower pH require the *pstII*-encoded transporter for sufficient phosphate uptake, possibly to secure phosphorous availability for oxidative phosphorylation coupled to aerobic respiratory electron transfer during iron oxidation, and/or to deal with poor solubility of phosphate arising from ferric iron sequestration at increasing pH. The ISAfe1 transposable insertion into the *pstII* operon affected the ability to oxidize iron, similarly to previously observed insertions in the *res* and *rus* operons in *Acidithiobacillus* spp. These mutations, and others yet to be described, seem to emerge frequently under permissive conditions (e.g. during growth on sulfur) only to reveal themselves upon the change in the growth mode.

## Conclusions

Combined nucleotide sequence, synteny, and gene complement comparative analyses of *A. ferrooxidans* strains proved to be a successful strategy to resolve subspecies-level taxa within the species. Discriminant genomic/genetic characteristics between sublineages included distinct flexible gene complements, MGEs repertoire, and differentiated MGE-associated gene cargo. Part of the differential gene complement may have become fixed in the respective sublineages for adaptive reasons. Adaptive genes linked to cell-environment interactions (e.g. glycosyltransferases, transporters) or to host cell-MGEs interactions (e.g. restriction-modification systems) were found in both sublineages 2A and 2B but differed in quality and/or quantity. How these gene functions relate to sublineages divergence requires further exploration.

Using *A. ferrooxidans* CCM 4253 as a test case, stability of both episomal and integrated MGEs under adaptive growth in both ferrous iron- and sulfur-containing media was observed, supporting their role in long-term adaptive processes. In turn, active replicative transposition of ISs (ISAfe1) after repeated culture transfers resulted in mutational inactivation of the *ptsC2* gene and impaired iron oxidation upon transfer to ferrous iron-containing media. These results support the previously observed phenomenon in stressed acidophiles and the role of ISs in short-term diversification under permissive conditions.

Impairment of growth of sulfur-adapted CCM 4253 cells in phosphate-rich media amended with iron as an energy source upon transfer (as reflected by the iron oxidation lag) revealed a role for the phosphate permease in the passive cytoplasmic acidification caused by the influx of dihydrogen phosphate and undissociated phosphoric acid in low pH medium.

## Methods

### Bacterial strains, growth conditions, treatments, and determinations

*A. ferrooxidans* was isolated from mine waters at Zlaté Hory in the Czech Republic and is deposited in the Czech Collection of Microorganisms (CCM) under number 4253. Bacterial strain CCM 4253 was cultivated onto overlay plates containing ferrous iron plus tetrathionate or tetrathionate only as electron donors^49^ at 30 °C. A single *A. ferrooxidans* CCM 4253 colony from the ferrous iron plus tetrathionate overlay plate was picked and multiplied in a basal salts medium containing ferrous iron at 30 °C on a rotary shaker. After sufficient cell numbers were achieved (1 × 10^8^ cells mL^–1^), the strain was cultured in basal salts media containing ferrous iron (9 g L^–1^ equivalent to 161 mM) or elemental sulfur (10 g L^–1^) as electron donors at 30 °C on a rotary shaker as described previously^48^. The long-term iron- and sulfur-adapted *A.* *ferrooxidans* CCM 4253 cultures were obtained after twenty transfers on the respective substrate (20 generations), as shown in Supplementary Fig. 2 (*Experiment 1*). Cells were harvested, and genomic DNA was obtained for resequencing.

The long-term ferrous iron-adapted culture (genotype *pstC2*) was transferred and passaged in a basal salts medium containing elemental sulfur. Cells were washed in a basal salts medium before being transferred to another substrate. The sulfur-oxidizing culture was cultivated at 30 **º**C on a rotary shaker until the pH dropped from an initial 3.5 to about 1.0 (10–14 days). Then 1/10 of the sulfur-grown culture was transferred to a fresh basal salts medium containing elemental sulfur and cultured under the same conditions. A total of six generations were prepared by this passaging, as shown in Supplementary Fig. 2 (*Experiment 2*). In addition, the long-term sulfur-adapted culture (genotype △*pstC2*) was transferred and passaged in a basal salts medium containing ferrous iron. Cells were washed in basal salts medium before being transferred to another substrate. The iron-oxidizing culture was cultivated at 30 °C on a rotary shaker until the complete ferrous iron was consumed (2–10 days). Then 1/10 of the iron-grown culture was transferred to a fresh basal salts medium containing ferrous iron and cultured under the same conditions. A total of six generations were prepared by this passaging, as shown in Supplementary Fig. 2 (*Experiment 3*). Aliquots of each short-term iron- and sulfur-adapted culture (1–6 generations) were harvested, genotyped, and phenotyped (ferric iron concentration and pH) as in *Experiment 1.* Ferric iron concentration was determined spectrophotometrically at 300 nm^50^. The pH values were measured using a Radiometer electrode and a laboratory pH meter PHM220 (MeterLab).

### DNA isolation and sequencing library preparation

Genomic DNA was isolated by phenol–chloroform extraction for next-generation sequencing techniques, as described earlier^51^, or using QIAamp® BiOstic® Bacteremia DNA Kit (Qiagen) for PCR applications. Plasmid DNA was isolated using the PureYield™ Plasmid Miniprep System (Promega). Extracted genomic DNA was purified on magnetic beads (KAPA Pure Beads, Roche) according to the standard protocol. The DNA concentration was measured using a Qubit ™ dsDNA HS Assay kit (Thermo Fisher Scientific). 150 ng of purified DNA was used to generate genomic libraries using the Kapa HyperPlus kit (Roche) with enzymatic fragmentation at 37 °C for 20 min. Adapter ligation was done using the SeqCap Adapter Kit A (Roche). Subsequently, the genomic libraries were again purified on magnetic beads, amplified, and re-purified on the same magnetic beads. Finally, both libraries were quantified using the KAPA Library Quantification Kit for Illumina® platforms. The preparation of genomic libraries proceeded according to protocol A, available in MiSeq System Denature and Dilute Libraries Guide (15039740v10). MiSeq Reagent Kit v2 for 300 cycles was used for sequencing, and the entire 151 bp paired-end sequencing system was prepared as described in the MiSeq System Guide (15027617v04). Genomic DNA obtained from *A. ferrooxidans* CCM 4253 was also commercially sequenced using Illumina technology (Macrogen, South Korea).

### Mitomycin C treatment and polymerase chain reactions

PCR validation of novel and partially conserved candidate iMGEs identified in the genome of strain CCM 4253 after treatment of *A. ferrooxidans* cells with mitomycin C, as previously described^23^. Amplification of target sequences was performed using GoTaq® DNA Polymerase (Promega) according to the protocol provided by the manufacturer. Oligonucleotides used in this study for PCR are listed in Supplementary Table 4E. The cycling conditions were as follows: initial denaturation for 15 min at 95 °C; 35 cycles consisting of denaturation for 30 s at 95 °C, primer annealing for 30 s at 60 °C, and extension for 40 s (*attLRPB*) or 2 min 20 s (*pstC2*) at 72 °C; followed by a final extension step for 7 min at 72 °C. PCR products were visualized on 1% agarose gels stained with GelRed® (Biotium).

### Genome sequencing and resequencing analysis

After quality control using FastQC and filtering using Trimmomatic^52^, sequence reads were assembled de novo using SPAdes genome assembler^53^. All contigs from mate-pair sequencing were aligned and ordered against the ATCC 23270^T^ genome using MAUVE^54^. The contigs positions were further confirmed by BLAST search against the assembly graph produced by SPAdes genome assembler from paired-end sequencing data. Contigs in the correct position and orientation were then manually linked. A total of 15 contigs were obtained. Non-overlapping gaps were filled by contigs produced by the SPAdes genome assembler. The complete chromosome and plasmid sequences were subsequently annotated using NCBI Prokaryotic Genome Annotation Pipeline (PGAP)^55^. Alignment and coverage analyses of iron- and sulfur-adapted *A. ferrooxidans* CCM 4253 phenotypes were made using Bowtie v1.2.2, and samtools v1.1 with default parameters, breadth and depth coverage were obtained using BBMAp v.38.94 and final files were upload to Figshare (see data availability).

### Genomes recovery from databases, gene-calling, and annotation

Genome drafts were obtained from NCBI (https://www.ncbi.nlm.nih.gov/assembly/) as of March 2020. We checked contamination and completeness as in Raes et al.^56^ and Manni et al.^57^. The resulting genome statistics are summarized in Supplementary Table 1B, along with sequence deposit information. Gene-calling and annotation were performed using the PGAP^55^. A genome sequence of low quality (AFE-DLC5, JNNH01) was annotated through the RAST pipeline (Rapid Annotation using Subsystem Technology)^58^. Recovered annotations were analyzed versus KEGG^82^ and COG^81^ databases as of July 2020 using SqueezeMeta^39^.

### Comparative genomics and synteny analysis

Overall genome relatedness indexes (OGRIs) and core, flexible, and exclusive genes were derived from Moya-Beltrán and colleagues^1^. All possible pairwise genome comparisons using the average nucleotide identities based on Blast (ANIb) or the in silico digital DNA–DNA hybridization index (dDDH) are summarized in Supplementary Table 3. Protein family clusters of predicted amino acid sequences of all open reading frames identified in the 2A and 2B sublineages strains are listed in Supplementary Table 5. Reciprocal percent identity of proteins conserved across species (*A. ferrooxidans* versus `*A. ferruginosus*´), across sublineages of *A. ferrooxidans* (2A versus 2B) or within each sublineage (2A versus 2A; 2B versus 2B) were assessed using GET_HOMOLOGUES software package v3.3.2^80^.

Draft genomes (contigs) were ordered against the *A. ferrooxidans* ATCC 23270 (CP001219.1) as reference genome using MAUVE version 2015-02-13 employing The Mauve Contig Mover (MCM). Ordered contigs were used to calculate the coverage percentage of strains against *A. ferrooxidans* ATCC 23270^T^ (sublineage 2A) and PQ505 (sublineage 2B) as references. Additional comparisons against related iron-oxidizing species were performed as indicated (*A. ferrianus* DSM 107098^T^, *A. ferridurans* ATCC 33020^T^, *A. ferrooxidans* ATCC 23270^T^, *A. ferriphilus* DSM100412^T^, *A. ferrivorans* DSM 22755^T^, and *'A. ferruginosus* CF3^T^'). Reconstruction and visualization of synteny blocks were done using SynChro^59^.

### Prediction and analysis of mobile genetic elements

The complete and high-quality draft genomes of *A. ferrooxidans* ATCC 23270^T^, ATCC 53993, and CCM 4253 (CP001219.1, NC_015850, CP001132.1) were used for the identification of putative MGEs using several programs such as ISfinder database^60^ and searched against the local database under conditions defined by 90% minimum similarity, 100% IS element coverage. ISEScan^61^ software was used for the de novo search of IS elements in chromosomal and TnpPred^62^ for predicting prokaryotic transposases. Manual curations of ISs and Tnp were performed to filter out false-positive results and incomplete IS elements. IslandPath^63^, AlienHunter^64^, and PAI-DA^65^ were used for genomic island identification, and Phage Finder^66^ and PhiSpy^67^ for prophage identification. CONJscan^68^ for Type IV secretion systems prediction and T346hunter software^69^ was used to identify conjugation genes, the Atlas T4SS^70^ database was searched, and the protein domains were identified using the CD search program. tRNAscan^71^ and Aragorn^72^ were used for tRNA and tmRNA searches. All predictions were analyzed and curated manually, as in Moya-Beltrán et al. 2019. Direct repeats were identified using the Needle program (EMBOSS) at the sequence termini. The BPROM^71^ program predicted bacterial promoters^73^. DnaA and IHF binding motifs were annotated manually, as in Chakravarty et al. 1995^43^.

### Data visualization and manipulation

Summary statistics and figures were computed using R packages: gdata v2.18.0, dplyr v1.0.2, plotly v4.9.0, ggplot2 v3.2.1, scales v1.0, RColorBrewer v1.1.2, readr v1.2.1, on Rbase v3.6.1 implemented in Rstudio v1.2.50001. Visualization of genome comparison by Blast was performed with Artemis Comparison Tool (ACT) v1.0. Figures of gene contexts and neighborhoods were performed with clinker v0.0.24^74^. Genome circular visualization was performed using Circos^75^.

## Supplementary Information

Supplementary Legends.

Supplementary Figure 1.

Supplementary Figure 2.

Supplementary Information 1.

Supplementary Information 2.

Supplementary Information 3.

Supplementary Information 4.

Supplementary Information 5.

Supplementary Information 6.

Supplementary Information 7.

## Data Availability

The datasets generated and/or analyzed during the current study are available from the National Center for Biotechnology Information (NCBI) Sequence-Read Archive (SRA) repository [https://www.ncbi.nlm.nih.gov/sra] under Biosample accessions: SAMN31181483 and SANN31181484. The Whole Genome Shotgun project of strain CCM 4253 has been deposited at GenBank under the accession number QKQP01. The version described in this paper is the first version, QKQP00000000.1. Other publicly available genomic sequences analyzed were downloaded from NCBI’s RefSeq FTP site. Supporting alignments in bam format and coverage values for each resequencing are available for download from Figshare at https://doi.org/10.6084/m9.figshare.20523591.
