# Supplementary material for: Influence of mobile genetic elements and insertion sequences in long- and short-term adaptive processes of Acidithiobacillus ferrooxidans strains
Source: Sci Rep. 2023 Jul 5;13:10876. doi: 10.1038/s41598-023-37341-4 (PMC10322971; doi:10.1038/s41598-023-37341-4)
Supplement: Supplementary file 1 — Supplementary Legends. [file 41598_2023_37341_MOESM1_ESM.docx]

**Supplementary Information File**

**Supplementary Table 1.** Strains and genomes used in this study and associated metadata. (A) Sequenced strains of *Acidithiobacillus ferrooxidans* were used in the study and metadata of origin. Data was recovered from listed publications, genome submission reports, and/or collected in this study. When not reported specifically in the literature, latitude, longitude, and altitude were calculated for the city, province, or prefecture available. The sample type was normalized as follows: water (water, drainage, acid mine drainage), slurry (mud, sediment), and soil (soil, ash). Genomes of strain assigned to *Acidithiobacillus ferrooxidans*, accession identifiers, and sequencing metadata. Data was recovered from genome submission reports, listed publications, and/or collected in this study. Sequencing and assembly methods and statistics are listed accordingly. The public genome of *A. ferrooxidans* ATCC 23270^T^ was used as a reference.

**Supplementary Table 2.** Relatedness of *Acidithiobacillus ferrooxidans* strains inferred from nucleotide data recovered from their sequenced genomes. (A) 16S rRNA gene sequence identities. 16S rRNA genes were recovered from the GenBank annotation of each of the genomes under comparison and their pairwise identities calculated using the BLASTn alignment algorithm, bidirectionally and averaged. (B) Average Nucleotide Identity based on Blast as alignment method (ANIb). (C) digital DNA:DNA hybridization (dDDH). Thresholds used for species delimitation are the following: digital DNA:DNA hybridization dDDH >70% (same genomic species [77,78]); Average Nucleotide Identity ANI >96% (same genomic species [76,79]). 16S rRNA clades 2A and 2B are defined in Nuñez et al., 2017 [2]. The public genomes of *Acidithiobacillia* class species type strains were used as references: *A. ferrianus* DSM 107098^T^, *A. ferridurans* ATCC 33020^T^, *A. ferrooxidans* ATCC 23270^T^, *A. ferriphilus* DSM100412^T^, *A. ferrivorans* DSM 22755^T,^ and *'A. ferruginosus* CF3^T^'. Type strains are marked with a T superscript.

**Supplementary Table 3.** Statistics of the pangenome analysis of *A. ferrooxidans* genomes pertaining to the 2A and 2B sublineages. Orthologs and protein families were obtained using GET_homologues [80] as described in Moya-Beltrán et al., 2021 [1]. (A) Protein family clusters occurrence and distribution in the 2A and 2B sublineage strains, according to their assignment to the pangenome gene complement pools (core, flexible and exclusive). (B) Reciprocal percent identity of proteins conserved across species (*A. ferrooxidans* versus `*A. ferruginosus*´), across sublineages of *A. ferrooxidans* (2A versus 2B) or within each sublineage (2A versus 2A; 2B versus 2B). (C) Number of protein families (presence-absence) (presence-absence) conserved between sublineages at different occurrence cut-offs (100% to 70% of all strains in each comparison type). (D) Functional assignment and classification of flexible and exclusive gene pool proteins using COG [81] and KEGG [82] databases.

**Supplementary Table 4.** iMGEs in *A. ferrooxidans* genomes. (**A**) Candidate and Validated iMGEs protein queries and their occurrence in the target *A. ferrooxidans* draft genomes using stringent (identity 90%; e-value E-10) and relaxed cut-offs (e-value E-10). (**B**) Extended gene vicinities identified in the target *A. ferrooxidans* draft genomes using iMGE-associated genes as search seeds, pangenome gene complements, and sub/lineages distribution profiles as filters. (**C**) Proteins of candidate iMGEs identified in the genome of *A. ferrooxidans* CCM 4253 (test case strain) and their occurrence in the target *A. ferrooxidans* draft genomes using stringent (identity 90%; e-value E-10) and relaxed cut-offs (e-value E-10). (**D**) The scheme shows the mobile genetic element's integrated (iMGE) and excised (eMGE) forms. Also shown is the empty chromosomal DNA (chDNA) site that remains after excision. The MGE and chDNA are shown as blue and red lines, respectively. The green boxes indicate the *attP, attL, attR, attP,* and *attB* sites. Oligonucleotides P1–4 used in PCR assays are shown as orange arrows above the target sequence. (**E**) Oligonucleotides used in this study for PCR validation of predicted iMGE occurence and location. (**F**) Original gel image in **Fig. 3D** at three different exposures.

**Supplementary Table 5.** Candidate eMGEs in the genomes of *A. ferrooxidans* strains. (A) List of all genes and protein products encoded in plasmid pAF4253 isolated from *A. ferrooxidans* CCM 4253. (B) Original gel image in **Fig. 4D** at three different exposures. (C) Summary of reads recovered from *A. ferrooxidans* CCM 4253 sequencing (QKQP01) and resequencing of iron- and sulfur-adapted cultures. Alignments in bam format and coverage values supporting the analysis (iron_seq_sorted.bam, sulfur_seq_sorted.bam, iron_seq_coverage.cov, and sulfur_seq_coverage.cov) are available at Figshare (doi: 10.6084/m9.figshare.20523591).

**Supplementary Table 6.** Functional assignment and classification of all recovered MGEs-associated gene products in *A. ferrooxidans* genomes. Genes are classified on the basis of their assignment to different pangenome gene complement pools, and presence /absence in the different sub/lineages under comparison, as well as by the CDD, COGs and KEGG classifications.

**Supplementary Table 7.** IS and RIT elements found in *A. ferrooxidans* CCM 4253. ISfinder database [60] and the ISEScan software [61] were used for the de novo search of IS elements in the genomic sequence of strain CCM 4253. The ISs were manually curated to filter out false-positive results, and incomplete IS elements. The final results include IS family, position in the genome (start-end and strand), gene ID, and IS annotation.

**Supplementary Figure 1.** Schematic workflow for recovery and reconstruction of MGEs from draft genomes. Steps are shown in black circles, resulting data is depicted by a representative scheme, and title and data repositories are represented as cylinders. Detailed procedures and tools used for candidate MGEs prediction can be found in Methods. Step 1: Genomes were recovered from the NCBI database (**Supplementary Table 1**). Step 2: Validated and candidate MGEs were identified from complete genomes, and protein-coding genes associated with each MGE were recovered (**Supplementary Table 6A**). Step 3: MGE-associated proteins were used as queries to search for candidate MGEs in complete and draft genomes of *A. ferrooxidans* strains. Both protein queries and hits identified in the target genomes were used to construct a protein repository of MGE-associated proteins in *A. ferrooxidans* **(Supplementary Table 6B**), with additional analyses performed. Step 4: Conserved, partially shared, and exclusive MGE-associated proteins were identified using pangenome analysis strategies. Step 5: MGE-associated genes were used as seeds to derive MGE gene vicinities in the target genomes, and pangenome gene complement classifications were used to identify MGE most likely limits (**Supplementary Table 6D**). Step 6: MGE-associated genes and their vicinities were analyzed using network reconstruction strategies to visualize MGE-associated functions in the genomes of the 2A and 2B sublineages (**Supplementary Table 6C**). Step 7: All information retrieved was used to identify novel MGEs in the *A. ferrooxidans* strain's draft genomes.

**Supplementary Figure 2.** The experimental design used to assess the effect of the electron donor or the switch between electron donors in MGE-mediated adaptation of *A. ferrooxidans* sublineage 2A strain CCM 4253. (**A**) *Experiment 1*: Genome resequencing of long-term iron and sulfur-adapted cultures. A single *A. ferrooxidans* colony from an overlay plate was picked and multiplied. The strain was cultured in basal salts media containing either ferrous iron or elemental sulfur. The long-term iron- and sulfur-adapted cultures were obtained after twenty transfers on the respective substrate (20 generations). Cells were harvested, and genomic DNA was obtained for resequencing using Illumina MiSeq technology. Read sequences obtained from long-term iron- and sulfur-adapted *A. ferrooxidans* CCM 4253 cultures were mapped to the assembled genome (QKQP01), and gross genomic changes were evaluated. The genotype of the derived cultures was compared to that of the culture of origin by PCR, directed to the *pstC2* gene sequence (**Supplementary Table 8**). Phenotyping of the cultures considered the presence/absence of growth, pH drop, and ferric iron production. (**B**) *Experiment 2*: Switching of long-term iron-adapted culture (genotype *pstC2*) to elemental sulfur (S^0^)-containing BSM. Aliquots of each short-term iron- and sulfur-adapted culture (1–6 generations) were harvested, genotyped by PCR to determine the coexistence of *pstC2* wild-type and △*pstC2* alleles, and phenotyped to determine the iron-oxidation lag phase as in *Experiment 1.* (**C**) *Experiment 3*: Switching of long-term sulfur-adapted culture (genotype △*pstC2*) to basal salts medium (BSM) containing Fe^2+^. (Figures were created with BioRender.com. (**D**) Full-length gels corresponding to the original gel displayed in **Fig. 6C** at three different exposures. (**E**) Full-length gels corresponding to the original gel displayed in **Fig. 6D** at three different exposures.
