# Supplementary figures and images for: Influence of mobile genetic elements and insertion sequences in long- and short-term adaptive processes of Acidithiobacillus ferrooxidans strains
Source: Sci Rep. 2023 Jul 5;13:10876. doi: 10.1038/s41598-023-37341-4 (PMC10322971; doi:10.1038/s41598-023-37341-4)

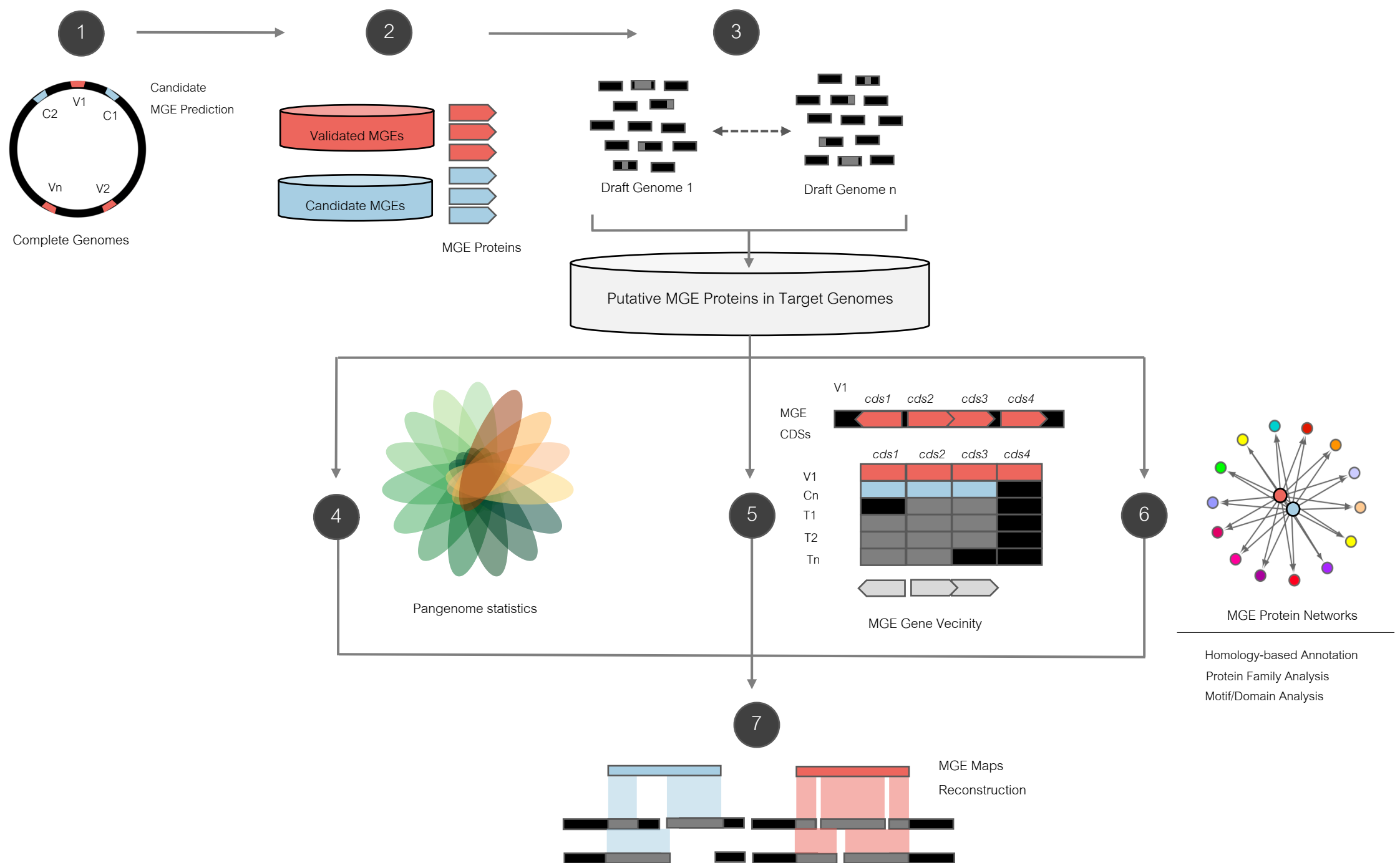

Supplement: Supplementary file 2 — Supplementary Figure 1. [file 41598_2023_37341_MOESM2_ESM.pdf]
