# Supplementary material for: Influence of mobile genetic elements and insertion sequences in long- and short-term adaptive processes of Acidithiobacillus ferrooxidans strains
Source: Sci Rep. 2023 Jul 5;13:10876. doi: 10.1038/s41598-023-37341-4 (PMC10322971; doi:10.1038/s41598-023-37341-4)
Supplement: Supplementary file 3 — Supplementary Figure 2. [file 41598_2023_37341_MOESM3_ESM.pdf]

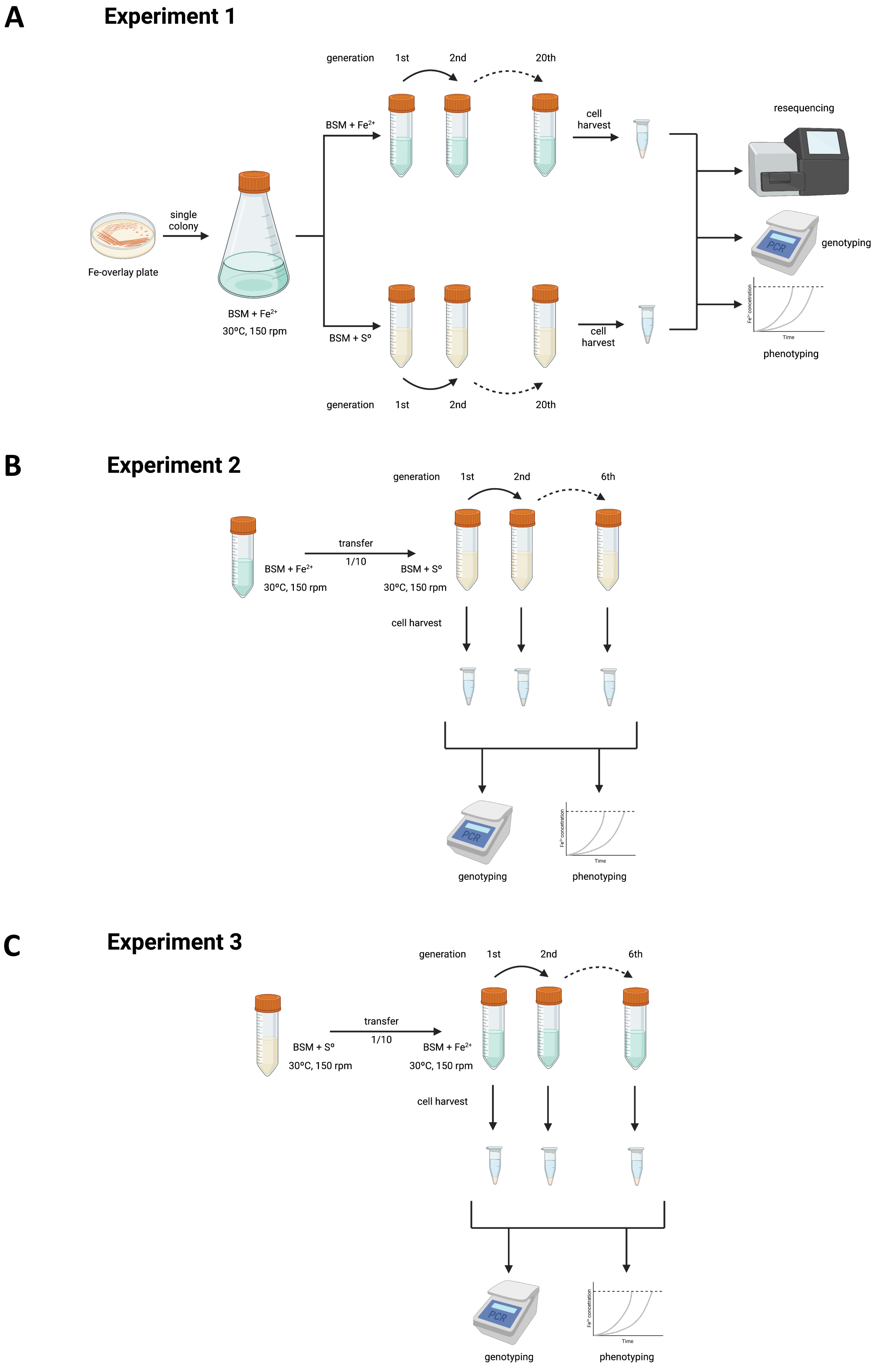
A
Experiment 1
generation
1st
2nd
20th
BSM + Fe^{2+}
cell harvest
resequencing
single colony
Fe-overlay plate
BSM + Fe^{2+}
30°C, 150 rpm
genotyping
BSM + S°
cell harvest
Time
phenotyping
generation
1st
2nd
20th
B
Experiment 2
generation
1st
2nd
6th
transfer
1/10
BSM + Fe^{2+}
30°C, 150 rpm
BSM + S°
30°C, 150 rpm
cell harvest
genotyping
phenotyping
C
Experiment 3
generation
1st
2nd
6th
transfer
1/10
BSM + S°
30°C, 150 rpm
BSM + Fe^{2+}
30°C, 150 rpm
cell harvest
genotyping
phenotyping

D

Exposure 1

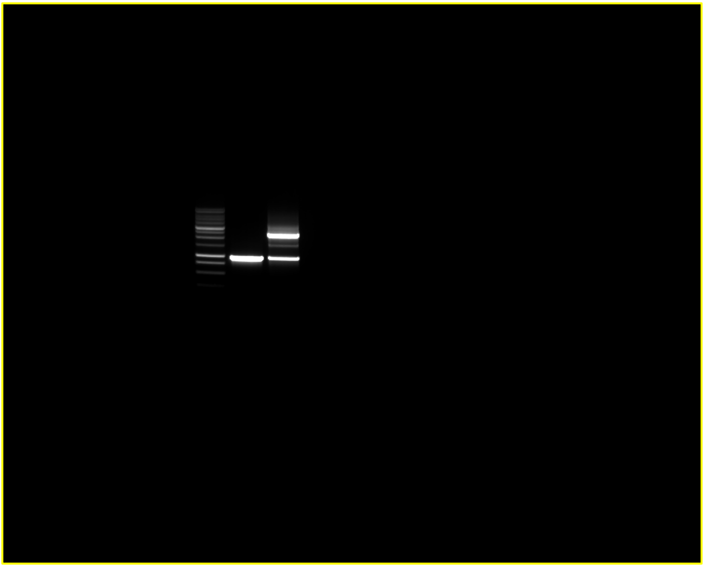

Exposure 2

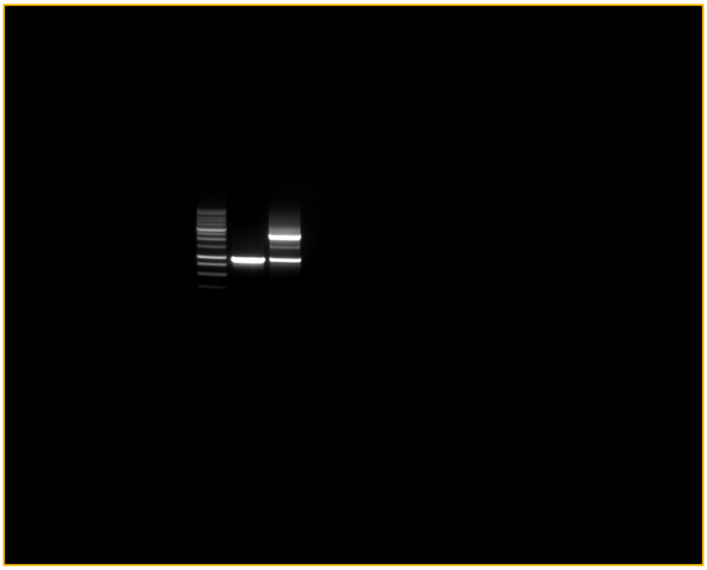

Exposure 3

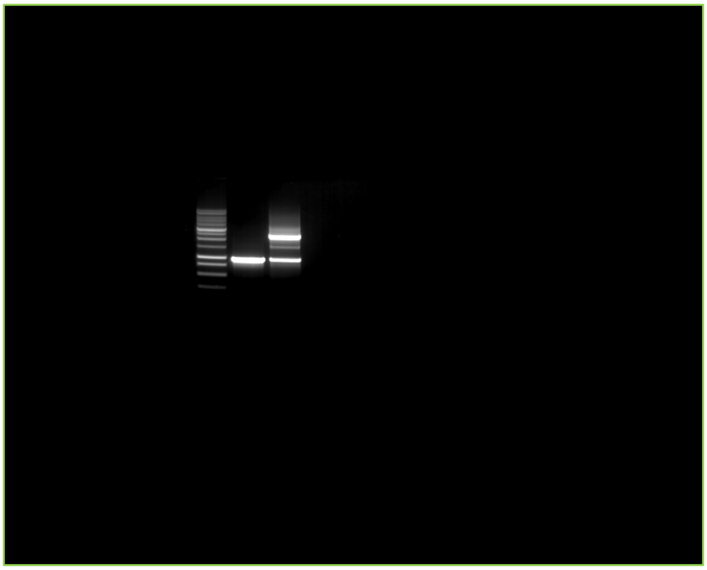

Original gel image at three different exposures for cropped gel in **Fig. 6C** corresponding to the PCR evaluation of the transpositional mutation in the long-term iron- and sulfur-adapted cultures (*Experiment 1*). Lane M represents a 1-kb DNA ladder; lane Fe^{2+} represents the PCR product of the *pstC2* locus from the iron-adapted culture; lane S^0 represents the PCR product of the *pstC2* locus from the sulfur-adapted culture.

E

Exposure 1

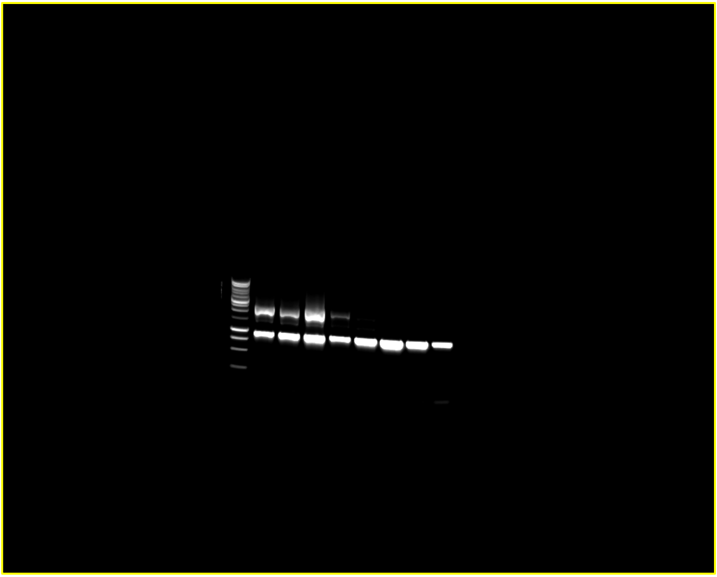

Exposure 2

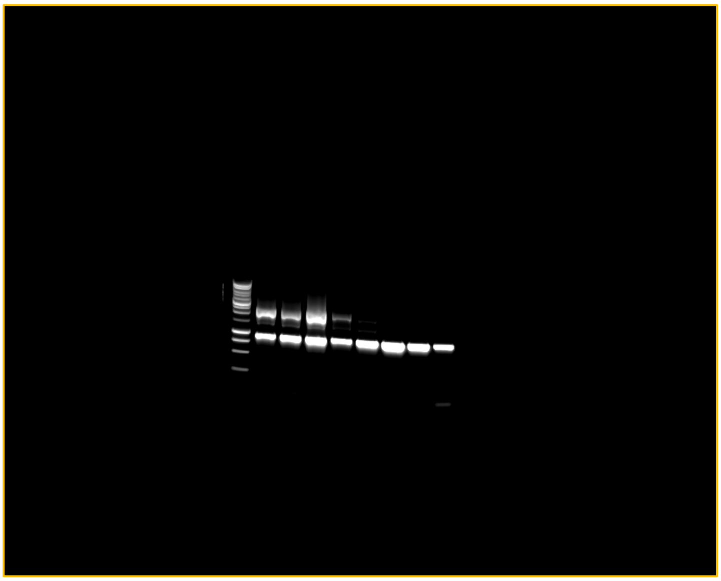

Exposure 3

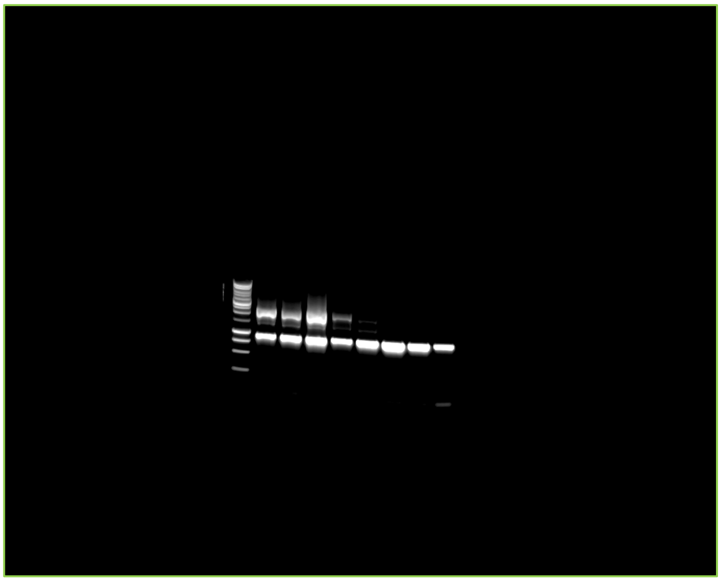

Original gel image at three different exposures for cropped gel in **Fig. 6D** corresponding to the PCR evaluation of reversal of the transpositional mutation during subsequent culture transfers (*Experiment 2*). Lane M represents a 1-kb DNA ladder; lanes 1–6 represent the PCR product of the *pstC2* locus from the first to sixth individual iron passages performed.
